# The Plagues of the Bible

**Published:** 1894-06-23

**Authors:** 


### The Plagues of the Bible.

It is difficult in these days for even Holy Writ to escape the inquiry of the scientist. This inquiry implies no irreverence. Many a one who, knowing how much there is of the inexplicable in life and nature, accepts without question the miracles recorded there, would fain have some clearer light on that magic of the Egyptians which Moses surpassed, the disease which killed that Herod who let his flatterers applaud his words as the voice of a God, and the thorn in the flesh which chastened the Apostle Paul. The sacred writers were historians and preachers, not specialists in any branch of science; moreover, they wrote for their own age as well as for ours, and sometimes used expressions which were perfectly clear to their contemporaries, but have little meaning for us. Thus the word "plague" which occurs so often in the record of God's dealings with His chosen people and their enemies, has exercised the curiosity and ingenuity of many doctors.

Plague, in the mediæval and modern sense, is a specific disease derived from the East, and thence called the Oriental or Levantine plague. It was specially characterised by pustules and carbuncles and easily recognised buboes of various glands. But plagues which are so often mentioned in the Old Testament do not seem invariably to have been of this nature. For one thing, this disease has not been proved to have existed before the second century of our era. This, however, means little; the disease may have been present long before, in the absence of any special knowledge, it was differentiated and diagnosed. This Oriental plague would account for many of the pestilences of Scripture; for example, that three days' of pestilence which was sent as a punishment to David for the murder of Uriah, when within seventy-two hours 70,000 people died. The same disease it may have been which worked such devastation when "the Assyrian came down like a wolf on the fold;" but the ravages of that visitation, by which, according to Josephus, "185,000 men with their captains and generals" were killed in a single night. So appalling and sudden is the destruction implied in this statement that various commentators on Josephus have suggested lightning or a sirocco as the agent. Herodotus, who alludes to the disaster, simply calls it a judicial disaster, and in the absence of clearer evidence than we possess, all conjecture must be of the most tentative nature.

One or two of the plagues we hear of seem to possess the characteristics of cholera. Such, for example, may have been the punishment that followed the eating of the quails by the wandering Israelites when they grew tired of manna, and longed for the flesh-pots of Egypt. The Bible narrative tells how a wind brought quails from the sea and let them fall by the camp, and for two days and a night the people gathered the quails, "and while the flesh was yet between their teeth, before it was chewed, the wrath of the Lord was kindled against the people, and the Lord smote the people with a very great plague." In view of the number of quails lying around the camp, decomposing under a tropical sun, it is easy to imagine that all the conditions for an epidemic of cholera were present, and a sudden change of food, greedily devoured, would greatly increase susceptibility to the disease. A Jewish rabbi once tried to show the character of the tenth plague of Egypt, which destroyed the first-born of all in the land, from the first-born of Pharaoh that sat on the throne, unto the first-born of the maid-servant, and all the first-born of beasts. But in this case it is impossible for a merely natural explanation of the plague to make clear how it came to exercise so strict a selection. Less unlikely is the suggestion that the boils and blains of the sixth plague were the symptoms of an epidemic of small-pox, though this, too, is a hazardous conjecture, regarding which one would hesitate to say yea or nay.

The disease which is most frequently mentioned by name in the Old Testament is leprosy. But with regard to this also there is much uncertainty. Leprosy to us means a certain bacillary disease, transmissable from the sick to the sound, and when once acquired quite incurable. It is evident that the leprosy of the Bible cannot all have been like this. Moses and Miriam had each brief attacks of leprosy as a punishment for sin, and after a period of isolation returned to the encampment cured. The leprosy of Naaman the Syrian was cured, though miraculously, by bathing in Jordan. The leprosy of Job, also, apparently a severe and repulsive disease, was yet cured, and the patriarch lived 140 years after his recovery. But in view of the fact that it is by no means certain that the Book of Job is to be regarded as historical, while it is clear that much of its language is to be regarded as metaphorical rather than literal, this case cannot be regarded as giving much evidence as to what the leprosy of the Bible was. The descriptions of the disease vary much. Sometimes it looks like leucoderma, sometimes like psoriasis; but it seems to have been always curable under treatment, the chief part of which was the strictest cleanliness. It was strictly a cutaneous disease, rather than a constitutional taint of the blood.

Other plagues are mentioned which we cannot associate with any known disease. Such is the plague with which, by the mouth of the prophet Zechariah, God threatened to punish all that fought against Jerusalem: "Their flesh shall consume away while they stand upon their feet, and their eyes shall consume away in their holes, and their tongue shall consume away in their mouth." Sir Risdon Bennett, who writes an interesting little book on the diseases of the Bible, surmises that this may mean the destruction of an army by famine—famine, which in war-time kills more than hostile swords. Other diseases were undoubtedly acquired by the children of Israel through their intercourse with the nations they were to supplant. Vices which are now hardly spoken of were then practised as religious rites, and the Israelites won to this shameful idolatry suffered for their sin. On one occasion Moses, in obedience to the divine command, killed all who were infected, to the number of 24,000. At a later period, when Israel was at war with the Midianites, Moses ordered the prisoners to be killed for fear of conveying the infection. Looking over the history of the Jews, with its frequent record of plague and pestilence, one is inclined to ask if all these visitations were indeed directly from the hand of God, and not, like our modern epidemics, the result of some neglect or defiance of the laws of health. Not less a divine visitation would they be for that, but a visitation intended to warn and instruct, not merely meant to punish.

